# Supplementary material for: Automated monitoring of tweets for early detection of the 2014 Ebola epidemic
Source: PLoS One. 2020 Mar 17;15(3):e0230322. doi: 10.1371/journal.pone.0230322 (PMC7077840; doi:10.1371/journal.pone.0230322)
Supplement: S1 Appendix — (DOCX) [file pone.0230322.s002.docx]

**Appendix**

1. **Architecture**

In this paper, we adapt an architecture for early detection of disease events using social media. The architecture is as follows:

1. The first step is ‘Initial Selection’ where tweets are selected based on location, date range and keywords. The keywords are words indicating a symptom. The location is derived based on either the tweet location, the author profile location or the location mention.
2. The second step is ‘personal health mention classification’. This step is necessary because a tweet containing a symptom word may not be the report of a symptom. The classifier uses tweet vectors as the feature representation for a statistical classification algorithm. A tweet vector is the average of word embeddings of content words in a tweet. The word embeddings used are pre-trained on a large corpus. A word embedding is a distributional representation of a word that is expected to capture the semantics of a word.
3. The third step is ‘duplication removal’. In this step, we retain the first tweet per day per user. This prevents multiple reports by the same user from swamping the system. It must be noted that this step follows the second step where a classifier has predicted a tweet as a health report.
4. The fourth step is ‘monitoring algorithm’. In this step, we use a monitoring algorithm based on time-between-events. The algorithm computes the expected duration between consecutive tweets. When the time between consecutive tweets is shorter than an expected value, the tweet is flagged. When p such consecutive tweets are flagged, an alert is generated.

In the original paper, the authors tested the architecture on a dataset of tweets posted in Melbourne. The architecture detected an alert 9 hours before the time in the official report.

1. **Location Parameters**

We use the following locations to search for our tweets:

1) Monrovia, Liberia [-118.001945 34.144260 40km]

2) Conakry, Guinea [-13.712550 9.509130 40km]

3) Freetown , Sierra Leone [-13.213670 8.482050 40km]

4) Gbarnga, Liberia [-9.473930 6.998350 40km]

5) Gueckedou, Guinea [-10.132300 8.559930 40km]

6) Kakata, Liberia [-10.349200 6.530090 40km]

7) Kenema , Sierra Leone [-10.061850 9.246780 40km]

8) Ganta, Libera [-8.986450 7.232230 40km]

We use [www.latlong.net](http://www.latlong.net) to obtain the latitude/longitude coordinates for these locations.

1. **Alerts for the classifiers**

For the personal health mention classification step, we experiment with two classifiers, namely SVM and SVM Perf. The alerts generated by the two classifiers are given in Table 1. We report SVM Perf in the main body of the paper.

|  | **SVM** | **SVM Perf** |
| --- | --- | --- |
| **Data Aggregation** | December 2013: 23, 24, 25, 26, 27  January 2014: 4, 5, 6, 7 ,8, 9, 10, 11  February – June 2014: None  July 2014: 4, 5, 6, 7, 8, 9, 22, 23, 25, 28, 29, 30, 31. | December 2013: 2, 4, 6, 7, 9, 10, 13, 14, 15, 16, 27, 28, 30  January 2014: 3, 4, 6, 10, 11, 13, 17, 18, 20, 24, 25, 27  February 2014: 21, 22, 23, 24, 28  March 2014: 1  April 2014: 24, 25  May 2014: 2, 3, 4, 5, 30  June 2014: 2, 5, 6, 7, 13, 14, 16, 18, 20, 21, 23, 24, 25  July 2014: 11, 12, 14, 15, 16,17, 18, 19, 21, 22, 23, 24, 25, 26, 27, 28, 29 |
| **Alert Aggregation (Union)** | December 2013: 9, 10, 16  January 2014: 5, 7, 8, 9  February-March 2014: None  April 2014: 8, 10, 11  May 2014: 28, 29  June 2014: None  July 2014: 3, 5 | December 2013: 26, 27, 28, 30  January 2014: 3, 4  February 2014: None  March 2014: 31  April 2014: 1, 27  May-June 2014: None |
| **Alert Aggregation (Intersection)** | None | 27^th^ December 2013 |

Table 1: Alerts generated by the two classifiers for the three adaptations

1. **EWMA charts for fever, rash and the data augmentation approach**

This section contains the EWMA charts based on counts as well as time between events. The red line is the line that signals less often with one of the following conditions: A false discovery rate of: (a) One in 2000 events, (b) One in 2000 days. The green line indicates the other condition.

## **Fever Counts**


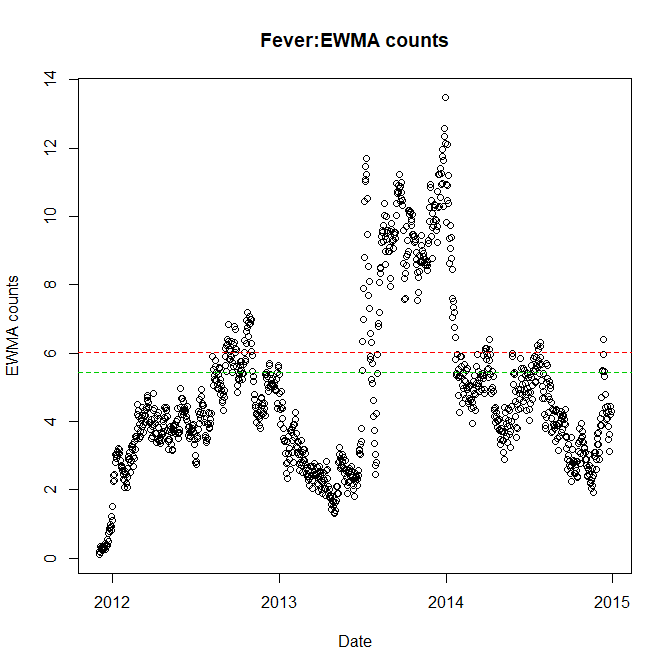


## **Rash Counts**


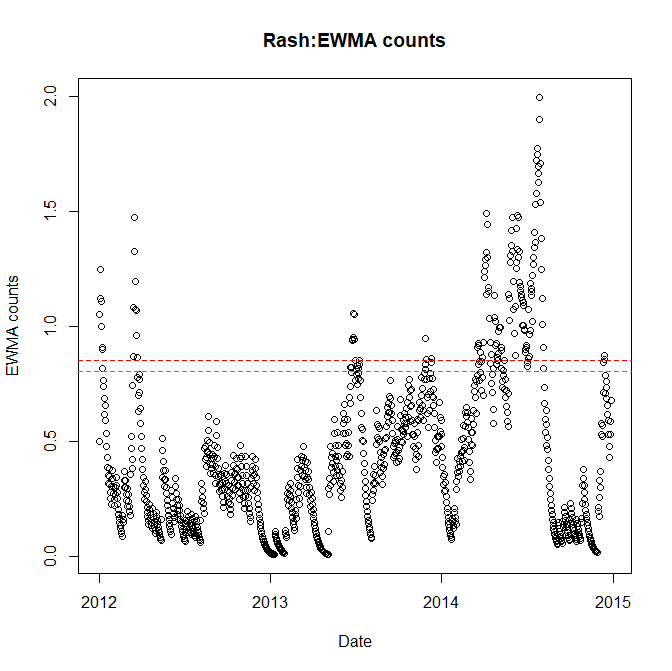


## **Joint fever and rash flags on the same day using personal health mentions counts**


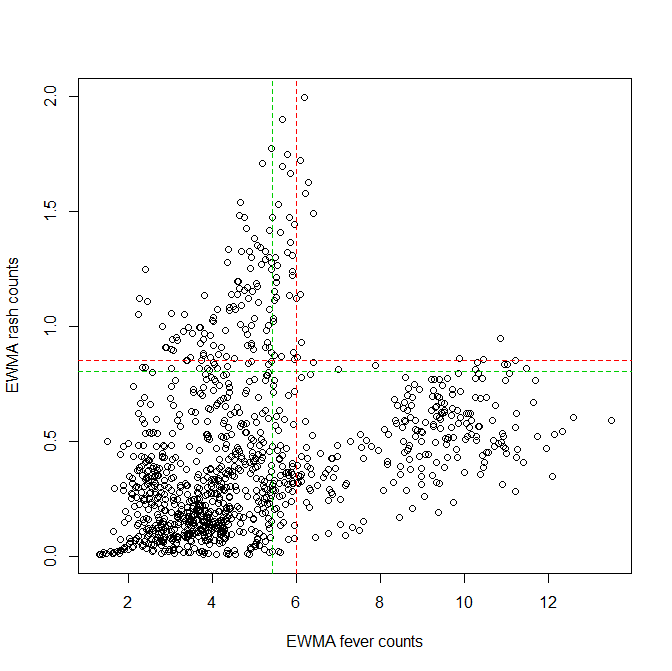


According to the count charts, the dates both signal greater than the threshold: are "2013-11-28" "2013-11-29" "2013-12-10" "2014-04-27" "2014-04-30" "2014-05-05" "2014-05-07"

The day-of-the-week for reporting times for fever time between events

Friday Monday Saturday Sunday Thursday Tuesday Wednesday

1234 1273 1135 421 478 495 441

We understand that the distribution of the symptom reports seems to be skewed. This points to a possibility that the data points may be biased. However, this chart is not based on duplicate removal or the consecutive alert filter in the monitoring step.

# **Time between events (TBE)**

## **TBE-based chart for rash**


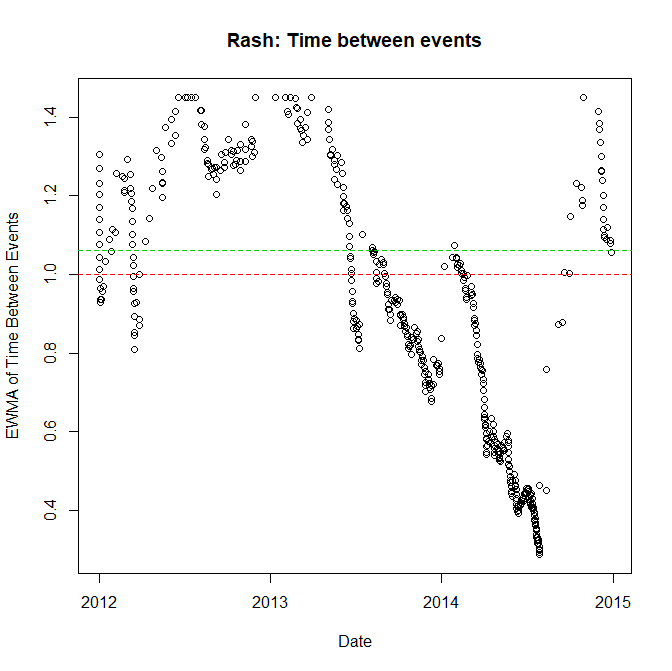


## **TBE-based chart for fever**


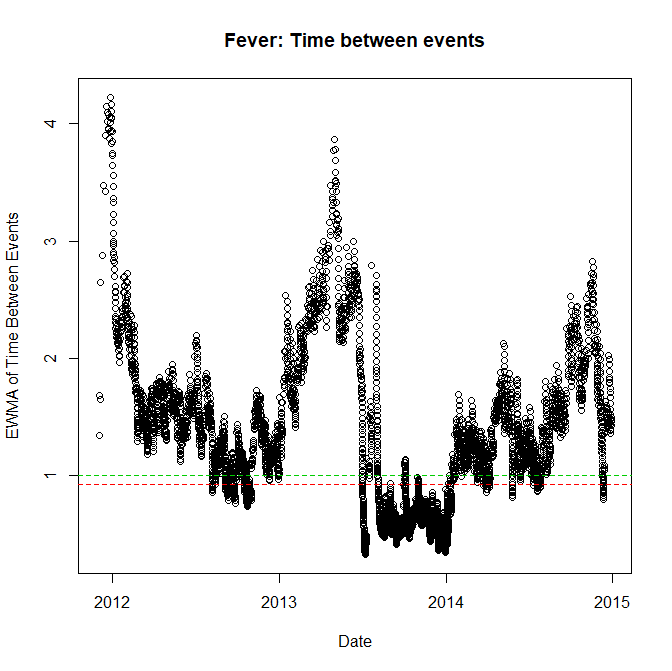


## **Joint fever and rash flags on the same day for time between events**

This chart is not based on duplicate removal or the consecutive alert filter in the monitoring step.


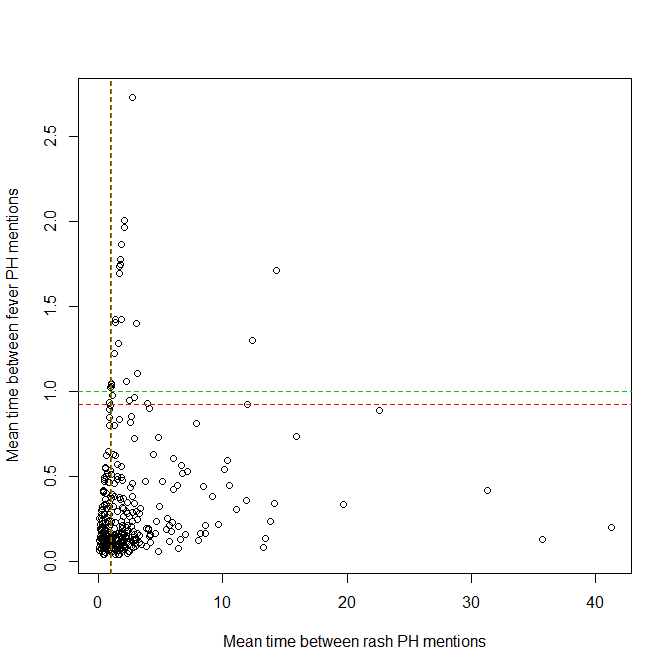


Joint signals on chart above

2012-01-01 2012-01-02 2012-01-03 2012-01-28 2012-03-08 2012-03-13 2012-03-14 2012-03-15

0.23337037 0.15839313 0.69448302 0.16681713 0.12471065 0.18754630 0.41182870 0.55563657

2012-03-16 2012-03-26 2012-05-14 2012-08-05 2012-08-06 2012-08-13 2012-08-18 2012-10-18

0.79904861 0.43412037 0.43585069 0.73049769 0.57703704 0.61410494 0.55078704 0.31593750

2012-10-28 2012-11-28 2013-03-10 2013-03-20 2013-05-04 2013-06-03 2013-06-04 2013-06-18

0.98308449 0.93291667 0.87299769 0.93532407 0.88782407 0.86967593 0.55273148 0.21856096

2013-06-22 2013-06-23 2013-06-24 2013-06-26 2013-06-27 2013-07-03 2013-07-06 2013-07-07

0.95259838 0.93243056 0.11990741 0.11881944 0.55324653 0.45192130 0.44349537 0.97228009

2013-07-09 2013-08-05 2013-08-14 2013-09-01 2013-09-06 2013-09-09 2013-10-13 2013-10-16

0.39908565 0.92800347 0.36940972 0.56737269 0.92252315 0.28805556 0.89408565 0.98001157

2013-10-22 2013-11-08 2013-11-13 2013-11-14 2013-11-18 2013-11-23 2013-11-24 2013-11-26

0.76304977 0.96538194 0.50236111 0.91526620 0.64349537 0.90309028 0.45039352 0.07627315

2013-12-06 2013-12-09 2013-12-21 2013-12-26 2014-02-03 2014-02-11 2014-02-20 2014-02-22

0.47181713 0.57757523 0.54028935 0.94474537 0.49079861 0.45564815 0.92823495 0.37509259

2014-03-09 2014-03-11 2014-03-16 2014-03-17 2014-03-18 2014-03-27 2014-03-31 2014-04-01

0.89317130 0.78184028 0.78699074 0.30298611 0.51783565 0.69644097 0.35871914 0.25051215

2014-04-03 2014-04-06 2014-04-07 2014-04-22 2014-04-23 2014-04-30 2014-05-03 2014-05-04

0.68392940 0.50022762 0.31483410 0.27913580 0.29738426 0.40344907 0.54972801 0.89846065

2014-05-05 2014-05-11 2014-05-12 2014-05-23 2014-05-24 2014-05-26 2014-05-27 2014-05-28

0.98543981 0.87462963 0.61773148 0.51581597 0.38254167 0.54116319 0.54021219 0.62381944

2014-05-29 2014-05-30 2014-06-07 2014-06-08 2014-06-10 2014-06-13 2014-06-18 2014-06-23

0.66721065 0.35386574 0.29544367 0.30435957 0.31796296 0.83119213 0.79437500 0.84190972

2014-06-24 2014-07-05 2014-07-08 2014-07-09 2014-07-10 2014-07-14 2014-07-15 2014-07-17

0.77733218 0.98994599 0.40457755 0.49545910 0.48673611 0.17209877 0.63353588 0.14755208

2014-07-18 2014-07-20 2014-07-21 2014-07-22 2014-07-23 2014-07-24 2014-07-26 2014-07-27

0.44028935 0.35748843 0.43626736 0.27445602 0.49083333 0.48087384 0.95582176 0.40912616

2014-07-28 2014-10-27 2014-12-03 2014-12-06 2014-12-07 2014-12-11 2014-12-12 2014-12-26

0.24488426 0.24521991 0.89614583 0.58103009 0.33514468 0.26305556 0.47591435 0.97125579

2014-12-28

0.77122685

IV: **Monitoring Algorithm (This is step 4 of the architecture)**

data<-read.table(”<filename>",sep="$")

data[1:4,]

#data<-data[,-2]

head(data)

data<-as.data.frame(data)

head(data)

data$V1<-as.Date(data$V1,format="%d.%m.%Y")

head(data)

data$yr <- as.numeric(as.vector(substring(data$V1,1,4)))

hr<-as.numeric(as.vector(substring(data$V2,1,2)))

min<-as.numeric(as.vector(substring(data$V2,4,5)))

sec<-as.numeric(as.vector(substring(data$V2,7,8)))

dtime<-hr+min/60+sec/3600

dtime[1:4]

data<-cbind(data,dtime)

wd<-weekdays(data$V1)

wd[1:4]

time<-as.numeric(as.vector(julian(data$V1)))

data<-cbind(data,time,wd)

min(data$time)

min(data$time)

summary(time)

data[is.na(data$time),]

data<-data[!is.na(data$time),]

mindatetime<-min(data$time)

data$time<-data$time-mindatetime

data$time<-data$time+dtime/24

data$time<-data$time+data$dtime/24

order(data$time)[1:1000]

dim(data)

sort(data$time)

ind<-order(data$time)

data<-data[ind,]

data$time

size <-length(data$time)

TBE<-data$time[-1]-data$time[-1*size]

summary(TBE)

data$time[-1]==data$time[-1*size]

TBE<-cbind(TBE,data[-1*size,])

temp<-TBE[TBE$TBE == 0,]

TBE = TBE[!TBE$TBE == 0,]

library(gamlss)

fm<-gamlss(TBE~time+as.factor(yr)*(cos(2*pi*time/365.25)+sin(2*pi*time/365.25))+wd*(dtime+cos(2*pi*dtime/24)+sin(2*pi*dtime/24)+cos(2*pi*dtime/12)+sin(2*pi*dtime/12)), sigma.fo=~time+as.factor(yr)*(cos(2*pi*time/365.25)+sin(2*pi*time/365.25))+wd*(dtime+cos(2*pi*dtime/24)+sin(2*pi*dtime/24)+cos(2*pi*dtime/12)+sin(2*pi*dtime/12)),data=TBE,family=WEI())

sigma <- exp(predict(fm, what=c("sigma"), newdata=temp))

mu <- exp(predict(fm, what=c("mu"), newdata=temp))

q <- 1:dim(temp)[1]/dim(TBE)[1]

temp$TBE = qWEI(p=q,mu=mu,sigma=sigma)

t4 = rbind(TBE, temp)

t4<-t4[order(t4$time),]

fm<-gamlss(TBE~time+as.factor(yr)*(cos(2*pi*time/365.25)+sin(2*pi*time/365.25))+wd*(dtime+cos(2*pi*dtime/24)+sin(2*pi*dtime/24)+cos(2*pi*dtime/12)+sin(2*pi*dtime/12)), sigma.fo=~time+as.factor(yr)*(cos(2*pi*time/365.25)+sin(2*pi*time/365.25))+wd*(dtime+cos(2*pi*dtime/24)+sin(2*pi*dtime/24)+cos(2*pi*dtime/12)+sin(2*pi*dtime/12)),data=t4,family=WEI())

SCALE<-fm$mu.fv

SHAPE<-fm$sigma.fv

scale<-fm$mu.fv[(length(SCALE)+1):length(fm$mu.fv)]

shape<-fm$sigma.fv[(length(SCALE)+1):length(fm$mu.fv)]

par(mfrow=c(1,1))

SCALE<-c(SCALE,scale)

SHAPE<-c(SHAPE,shape)

TBE<-t4

h<-rep(0,length(SCALE))

#BETA<-read.csv("//Users//jos059//ThunderstormAsthma//TBE.ANE1000.BETAS.csv", header=TRUE)

#alsq = BETA$al^2

#alcub = BETA$al^3

#shapsq = BETA$shape^2

#shapcub = BETA$shap^3

#al = BETA$al

#BETA2 <- cbind(BETA,alsq,alcub,shapsq,shapcub)

#fm2<- lm(formula = beta1 ~ al + shape + log(al)+ log(shape) + alsq + alcub + shapsq + shapcub + al * (shape + log(shape) + shapsq + shapcub) + log(al) * (shape + log(shape) + shapsq + shapcub) + alsq * shapsq + shape * alsq + shapcub * alcub, data = BETA2)

#BETA2<-cbind(BETA2, newbeta)

#head(BETA2)

x.mu<-SCALE*gamma((1/SHAPE)+1)

#ind<-rank(c(SHAPE[1],BETA2$shape[BETA2$al==0.05]))[1]

#ind<-as.integer(ind)

#BETAT<-BETA2[BETA2$al==0.05,][ind,]

#ALL<-unique(BETA2$al)

ew<-x.mu

xmu<-SCALE[1]

al<-0.05

newbeta=15.4863461-31.2740388*al-22.978514*SHAPE[1]+6.1326295*log(al)+ 9.3864181*log(SHAPE[1])+ 8.1190440 *al^2 -14.7686294*al^3 +9.9260160*SHAPE[1]^2 -1.9660931*SHAPE[1]^3 +42.3619299*al*SHAPE[1]-17.5618988 *al*log(SHAPE[1]) -17.2181503 *al*SHAPE[1]^2+ 3.5450699*al*SHAPE[1]^3-9.5183478*SHAPE[1]*log(al)+3.8567301*log(al)*log(SHAPE[1])+4.0985140*log(al)*SHAPE[1]^2-0.8025912*log(al)*SHAPE[1]^3 -6.4936384*al^2*SHAPE[1]^2+6.4764153* SHAPE[1]*al^2+ 3.436274*al^3*SHAPE[1]^3

h[1]<-newbeta*SCALE[1]

xmu<-min(0.1*TBE$TBE[1]/gamma((1/SHAPE[1])+0.9*xmu),SCALE[1])

ew[1]<-min(c(al*TBE$TBE[1]/h[1]+(1-al)*x.mu[1]/h[1],x.mu[1]/h[1]))

ew.last<-ew[1]

for(I in 2:length(SHAPE)-1){

alopt<-(-0.32699401+0.02981035*xmu+0.27631529*scale+0.03879301*shape-0.17932719*log(xmu)+0.0882661*log(scale)+0.01238951*xmu*scale- 0.06672816*xmu*shape+0.17150974* xmu*log(xmu)+0.04734040*scale*shape+0.07158034*scale*log(xmu)-0.02679079*shape*log(xmu)-0.09427231*scale*log(scale)-0.29939506*xmu*log(scale)-0.01674911* log(xmu)*log(scale))

alopt[alopt<0.03]<-0.03

alopt[alopt>0.25]<-0.25

al<-max(c(0.02,alopt),na.rm=T)

al<-min(c(0.25,al),na.rm=T)

newbeta=15.4863461-31.2740388*al-22.978514*SHAPE[I]+6.1326295*log(al)+ 9.3864181*log(SHAPE[I])+ 8.1190440 *al^2 -14.7686294*al^3 +9.9260160*SHAPE[I]^2 -1.9660931*SHAPE[I]^3 +42.3619299*al*SHAPE[I]-17.5618988 *al*log(SHAPE[I]) -17.2181503 *al*SHAPE[I]^2+ 3.5450699*al*SHAPE[I]^3-9.5183478*SHAPE[I]*log(al)+3.8567301*log(al)*log(SHAPE[I])+4.0985140*log(al)*SHAPE[I]^2-0.8025912*log(al)*SHAPE[I]^3 -6.4936384*al^2*SHAPE[I]^2+6.4764153* SHAPE[I]*al^2+ 3.436274*al^3*SHAPE[I]^3

h[I]<-newbeta*SCALE[I]

ew[I] <- min(c(al*TBE$TBE[I]/h[I]+(1-al)*ew.last,x.mu[I]/h[I]))

if(ew[I]<1)ew.last<-x.mu[I]/h[I] else ew.last<-ew[I-1]

xmu<-min(0.1*TBE$TBE[I]/gamma((1/SHAPE[I])+1)+0.9*xmu,SCALE[I])

}

plot(TBE$V1,ew[1:length(TBE$V1)],xlab="Date",ylab="Adaptive EWMA")

abline(h=1,col=2)

ew2<-ew<1

ewint <- ew2[1:length(TBE$V1)]

ewres <- numeric(length(TBE$V1))

for (i in 8:length(ewint)) {ewres[i] <- ewint[i-1] + ewint[i-2] + ewint[i-3] + ewint[i-4] + ewint[i-5] + ewint[i-6] + ewint[i-7]}

ew2<-ew<

plot(TBE$V1[8:length(TBE$V1)], ewres[8:length(TBE$V1)], xlab="Date", ylab="Number of Consecutive Alerts")

abline(h=4, col=2)

allops <- cbind(TBE,ewres)

alerts<-allops[allops$ewres >= 4,]

length(alerts$V1)
