# Supplementary material for: Automated monitoring of tweets for early detection of the 2014 Ebola epidemic
Source: PLoS One. 2020 Mar 17;15(3):e0230322. doi: 10.1371/journal.pone.0230322 (PMC7077840; doi:10.1371/journal.pone.0230322)
Supplement: Attachment — Submitted filename: PONE-D-19-27142-ResponseDocument.docx [file pone.0230322.s003.docx]

| **Reviewer Comments** | **Author Response** |
| --- | --- |
| Please ensure that your manuscript meets PLOS ONE's style requirements, including those for file naming. The PLOS ONE style templates can be found at  <http://www.journals.plos.org/plosone/s/file?id=wjVg/PLOSOne_formatting_sample_main_body.pdf> and <http://www.journals.plos.org/plosone/s/file?id=ba62/PLOSOne_formatting_sample_title_authors_affiliations.pdf> | We have created the title page as indicated. |
| 1. Thank you for including your funding statement; "The funders had no role in study design, data collection and analysis, decision to publish, or preparation of the manuscript."   a. Please provide an amended Funding Statement that declares *all* the funding or sources of support received during this specific study (whether external or internal to your organization) as detailed online in our guide for authors at <http://journals.plos.org/plosone/s/submit-now>. b. Please state what role the funders took in the study.  If any authors received a salary from any of your funders, please state which authors and which funder. If the funders had no role, please state: "The funders had no role in study design, data collection and analysis, decision to publish, or preparation of the manuscript.   Please include your amended statements within your cover letter; we will change the online submission form on your behalf. | We have stated the funding statement in the cover letter:  "There are no third-party funders with conflict of interest for this project. This research is funded internally by the employers of the paper." |
| 2. We note that you have indicated that data from this study are available upon request. PLOS only allows data to be available upon request if there are legal or ethical restrictions on sharing data publicly. For information on unacceptable data access restrictions, please see <http://journals.plos.org/plosone/s/data-availability#loc-unacceptable-data-access-restrictions>. | This has been addressed in the next row. |
| In your revised cover letter, please address the following prompts:  a) If there are ethical or legal restrictions on sharing a de-identified data set, please explain them in detail (e.g., data contain potentially identifying or sensitive patient information) and who has imposed them (e.g., an ethics committee). Please also provide contact information for a data access committee, ethics committee, or other institutional body to which data requests may be sent. |  |
| b) If there are no restrictions, please upload the minimal anonymized data set necessary to replicate your study findings as either Supporting Information files or to a stable, public repository and provide us with the relevant URLs, DOIs, or accession numbers. Please see <http://www.bmj.com/content/340/bmj.c181.long> for guidelines on how to de-identify and prepare clinical data for publication. For a list of acceptable repositories, please see <http://journals.plos.org/plosone/s/data-availability#loc-recommended-repositories>.  We will update your Data Availability statement on your behalf to reflect the information you provide. | Along with this submission, we have uploaded the dataset files as supporting information files. The files contain tweet identifiers as allowed by Twitter’s data sharing policy.  This dataset will be hosted on CSIRO Data Access Portal and will have a DOI.  We have addressed specific reviewer comments pertaining to this below. |
| [Note: HTML markup is below. Please do not edit.]  Reviewers' comments:  Reviewer's Responses to Questions  **Comments to the Author**  1. Is the manuscript technically sound, and do the data support the conclusions?  The manuscript must describe a technically sound piece of scientific research with data that supports the conclusions. Experiments must have been conducted rigorously, with appropriate controls, replication, and sample sizes. The conclusions must be drawn appropriately based on the data presented.   Reviewer #1: Yes  Reviewer #2: Yes | Thank you for this. |
| 2. Has the statistical analysis been performed appropriately and rigorously?   Reviewer #1: N/A  Reviewer #2: Yes | Thank you for this. |
| 3. Have the authors made all data underlying the findings in their manuscript fully available?  The [PLOS Data policy](http://www.plosone.org/static/policies.action#sharing) requires authors to make all data underlying the findings described in their manuscript fully available without restriction, with rare exception (please refer to the Data Availability Statement in the manuscript PDF file). The data should be provided as part of the manuscript or its supporting information, or deposited to a public repository. For example, in addition to summary statistics, the data points behind means, medians and variance measures should be available. If there are restrictions on publicly sharing data—e.g. participant privacy or use of data from a third party—those must be specified.  Reviewer #1: Yes  Reviewer #2: No | We have addressed the concern of Reviewer 2, against their textual comments in a row below. |
| 4. Is the manuscript presented in an intelligible fashion and written in standard English?  PLOS ONE does not copyedit accepted manuscripts, so the language in submitted articles must be clear, correct, and unambiguous. Any typographical or grammatical errors should be corrected at revision, so please note any specific errors here.  Reviewer #1: Yes  Reviewer #2: No | We have addressed the concerns of reviewer #2, against their textual comments in a row below. |
| 5. Review Comments to the Author  Please use the space provided to explain your answers to the questions above. You may also include additional comments for the author, including concerns about dual publication, research ethics, or publication ethics. (Please upload your review as an attachment if it exceeds 20,000 characters) | - |
| **Reviewer #1** | |
| Reviewer #1: This is a very interesting article where the investigators used datasets derived from social media (tweets) and applied two adaptations of an existing surveillance architecture with automated monitoring to test for early detection of the 2014 Ebola epidemic. Using this social media-based monitoring, an early alert was detected in December 2013, three months before the official announcement. The authors took into account the probability and relevance of a symptom, its seasonal appearance and its occurrence over time, which are important in terms of specificity to the disease, when trying to combine symptom reports to detect a disease alert in social media. | Thank you for the summary. |
| Comments: 1. The sentence at the end of the abstract “An additional observation of relevance to infectious disease surveillance in general, is regarding the second adaptation which produces a restricted but useful set of alerts” could be edited in order to convey the message more strongly. It could be rephrased as “The second adaptation, which produces a restricted but useful set of alerts, could potentially be applied in other infectious disease surveillance and alert systems” | We have updated the last sentence of the abstract as indicated in the comment:  “The second adaptation, which produces a restricted but useful set of alerts, could potentially be applied in other infectious disease surveillance and alert systems” |
| 2. The authors correctly report that the symptoms they searched for were fever and rash. However, they do not adequately explain why they have not tried to incorporate other (than muscle pain) symptoms of ebola virus disease, like headache, diarrhea, vomiting or bleeding in the architecture. | We have updated the description to include the following text:  “We experimented with bleeding and red eyes but the number of tweets obtained were few. Therefore, as a simplistic setting, we stick to two symptoms: fever and rash. This also makes the union and intersection operations simpler. While the architecture could be applied to more than two symptoms in principle, we do not show the results for the same in this paper because we stick to only two symptoms of Ebola.” |
| 3. The authors could discuss in more detail if and how their model is applicable in other social media platforms or other (rare or common) diseases. | To address the comment, we have added a paragraph to the end of the conclusion section (now renamed to ‘Conclusion and future work’):  “As future work, our architecture could be adapted for other social media platforms or disease types. Our choice of Twitter as a social media platform is due to the availability of its API and access to Twitter data for research purposes. The architecture may be applicable to other social media platforms with usage frequency and content similar to Twitter. In other words, the architecture may need to be modified for a social media platform that gets used less frequently or has longer posts. Also, we apply the architecture for Ebola in this paper. This could be applied to unexpected diseases such as acute disease events, or common diseases such as influenza.” |
| **Reviewer #2** | |
| Reviewer #2: In the paper “Automated monitoring of tweets for early detection of the 2014 Ebola epidemic” the authors present two methods for analyzing the language in social media posts (specifically Twitter “tweets”) to give epidemic alerts. These two methods are described as adaptations of an architecture which has just been accepted for publication elsewhere (the authors citation number 8). Using a data set of tweets from between 2011 and 2014 the authors are able to predict the Ebola epidemic in West Africa three months prior to what they call the official announcement of the epidemic. They do this by analyzing the frequency of the use of words relevant to a particular disease. | Thank you for the summary. |
| I find the subject matter of the paper interesting and important, however I do not feel like the authors have done a sufficient job explaining why their approach is different or better from other methods being used in the literature. Although I find it likely that the method presented in the paper improves upon their previous architecture, I would have appreciated it if the need for improvement was addressed. The performance between these two architectures was discussed, but a performance comparison with their previous architecture for this particular data set was not mentioned. | 1) To explain why our approach is better, we rephrased the following paragraph and also made some additions to its previous version:  “However, past work monitors several symptoms of an illness as a collection, and constructs a stream of tweets that is then monitored. For example, for detection of influenza, tweets containing reports of cough, cold, fever, etc. may be put together. However, different symptoms have different prior probabilities of being reported (for example, a fever may be more common than rash or a fever may be reported with less stigma as compared to rash) and different seasonal appearances (for example, cold may be more common in winters while dehydration may be more common in summers). Therefore, tweets reporting each of these symptoms may have different time series behaviours in terms of magnitudes and trends. In this regard, our paper differs from past work in social media-based infectious disease surveillance.”  2) To address the concern about previous architecture, we added the following sentence:  “Since steps 2, 3 and 4 are the same as the base 4-step architecture, data aggregation closely resembles past architecture.” |
| Unfortunately the data used is not publicly available, since a premium twitter API requires that the user pay a monthly fee, and so I have to recommend rejection, although I do believe that after some editing the paper deserves to be published, just not in a public library of science journal. | The creation of a similar dataset would require access to the Twitter Premium API. However, downloading tweets using their tweet identifiers does not require a monetary subscription at the time of drafting this response document.  As per Twitter’s data sharing policy, we are able to share the identifiers of tweets. Therefore, we have uploaded the dataset files as supplementary information files. We will also host them to a repository with a DOI.  A twitter developer ID can be used to access the text and the timestamps of these tweets: two attributes required for steps 2 and 3 of our architecture. |
| PLOS One has seven criteria for publication which I will address in turn.  1. “The study presents the results of primary scientific research”. The authors present a comparison of two novel architectures on a data set of tweets, and so is a paper describing a new methodology, and does meet PLOS One’s crieteria for “primary scientific research”. | Thank you for your observation. |
| 2. “Results reported have not been published elsewhere”. As far as I can tell this is the case. | Thank you for your observation. |
| 3. “Experiments, statistics, and other analyses are performed to a high technical standard and are described in sufficient detail”. Other papers that aim to use social media posts to predict disease often have more technical statistical methods for analysis, or are implementing more standard methods of data sorting for prediction, such as Decision Trees and Random Decision Forests. The lack of such methodology is not a failing of the paper, I assume that this is one of the merits of the methodology. If it works about as well, or maybe even better, but is simpler to understand and implement, then it qualifies as preferable by those standards. If that is the case I think that it should be highlighted, and other works should be cited for comparison. | 1) Comparison with other methods for prediction  We choose support vector machines as the classifier training algorithms because they have shown to perform better than decision trees and random forests for personal health mention classification, as reported in the paper by:  Aramaki, Eiji, Sachiko Maskawa, and Mizuki Morita. "Twitter catches the flu: detecting influenza epidemics using Twitter." In Proceedings of the conference on empirical methods in natural language processing, pp. 1568-1576. Association for Computational Linguistics, 2011.  We have added the text above to the draft. |
| I think that the four step method deserves a bit more detail. For instance, “The algorithm computes the expected duration between consecutive tweets. When the time between consecutive tweets is shorter than an expected value, the tweet is flagged”. Although I can imagine how this is being done, I do not find it clearly stated how it is done. | The reviewer seems to refer to Step 4 of the method in the quoted text:  “Time-between-events corresponds to the duration between consecutive events in a time series. Of relevance to our algorithm is the event of a tweet being posted. Using in-control data, the algorithm fits a Weibull distribution and estimates its parameters. During test time, the algorithm computes the expected duration between times of posting for consecutive tweets. When the time between consecutive tweets is shorter than an expected value, the tweet is flagged. When p such consecutive tweets are flagged, an alert is generated. The detailed code of the monitoring algorithm has been implemented in R, and is included in the appendix.”  For step 2, we add the sentence: “The classification step uses support vector machines trained on a labelled dataset where each tweet is represented as its tweet vector.”  For step 1, we update the last sentence as follows: “Please note that twitter authorisation keys to access the API have been removed since they are personal to this author.”  We had omitted this information because they are described in detail in the original paper. |
| 4. “Conclusions are presented in an appropriate fashion and are supported by the data”. The conclusions stated seem to be factual. | Thank you for your observation. |
| 5. “The article is presented in an intelligible fashion and is written in standard English”. There are instances in which the grammar needs to be fixed. As I understand it, there is no copy editor, and so the authors must do this themselves. As an example, “Most past work in social media-based infectious disease surveillance uses datasets created using a set of symptom words. before official health systems detect it.”, which appears in the third paragraph of the introduction. This certainly hurts the readability, and for this sentence in particular, does not qualify as intelligible. Other than the overt mistakes, the paper is intelligible. | We have changed this to “The goal is to signal a disease outbreak before official health systems detect it.”  In addition, we also did a thorough proofreading of the paper to correct overt mistakes, as pointed out by the reviewer. Specifically,  1) However, our claim is that a change in the prevalence of symptom reports on social media can be an indicator of an outbreak, and can supplement infectious disease surveillance.: “and can supplement infectious disease surveillance” added.  2) “For example, for detection of influenza,”: “, for” added.  3) This holds true for all versions of our architecture.: ‘the’ removed. |
| 6. “The research meets all applicable standards for the ethics of experimentation and research integrity”. I see no issues. | Thank you for your observation. |
| 7. “The article adheres to appropriate reporting guidelines and community standards for data availability”. It is reported here that “For our experiments, we created a dataset of tweets. We used the Twitter Premium Search API…”. Twitter premium search API has a monthly fee associated with it, and so fails to meet the criteria of PLOS journals “PLOS journals require authors to make all data underlying the findings described in their manuscript fully available without restriction at the time of publication”. Having to pay for the data is a restriction. | As stated above, we have uploaded files containing the unique identifiers of the tweets. Using a Twitter developer API (with requisite permissions from Twitter, none of which require payment), the text and timestamps can be downloaded as required in steps 2 and 3 of the architecture. |
| 6. PLOS authors have the option to publish the peer review history of their article ([what does this mean?](https://journals.plos.org/plosone/s/editorial-and-peer-review-process#loc-peer-review-history)). If published, this will include your full peer review and any attached files.   If you choose “no”, your identity will remain anonymous but your review may still be made public.   **Do you want your identity to be public for this peer review?** For information about this choice, including consent withdrawal, please see our [Privacy Policy](https://www.plos.org/privacy-policy).  Reviewer #1: No  Reviewer #2: No | - |
| [NOTE: If reviewer comments were submitted as an attachment file, they will be attached to this email and accessible via the submission site. Please log into your account, locate the manuscript record, and check for the action link "View Attachments". If this link does not appear, there are no attachment files to be viewed.]   While revising your submission, please upload your figure files to the Preflight Analysis and Conversion Engine (PACE) digital diagnostic tool, <https://pacev2.apexcovantage.com/>. PACE helps ensure that figures meet PLOS requirements. To use PACE, you must first register as a user. Registration is free. Then, login and navigate to the UPLOAD tab, where you will find detailed instructions on how to use the tool. If you encounter any issues or have any questions when using PACE, please email us at [figures@plos.org](mailto:figures@plos.org). Please note that Supporting Information files do not need this step. | - |
